# Editorial: Statistical and computational methods for single-cell sequencing analysis

**DOI:** 10.3389/fgene.2023.1235174

**Published:** 2023-06-21

**Authors:** Lin Hou, Zhicheng Ji, Jingshu Wang, Jichun Xie

**Affiliations:** ^1^ Center for Statistical Science, Tsinghua University, Beijing, China; ^2^ Department of Biostatistics and Bioinformatics, Duke University, Durham, NC, United States; ^3^ Department of Statistics, University of Chicago, Chicago, IL, United States; ^4^ Department of Mathematics, Duke University, Durham, NC, United States

**Keywords:** single-cell sequencing, single-cell ATAC sequencing, integrative analysis, methods benchmark, clustering

Single-cell sequencing technologies, adopted extensively over the past decade, generate voluminous data that profile a myriad of cellular features. To harness the full potential of single-cell sequencing, it is crucial to develop powerful, efficient, and robust computational and statistical methods for analyzing the data and extracting meaningful insights. This Research Topic showcases five seminal papers that introduce novel analytical techniques and benchmark existing methods in this domain.

One of the key areas of focus in recent years has been the analysis of single-cell ATAC sequencing (scATAC-seq) data. ScATAC-seq data measure chromatin accessibility in individual cells. Sophisticated statistical and computational techniques are required to analyze scATAC-seq data, specifically for clustering and trajectory reconstruction analysis, a common requirement for scRNA-seq data. The paper titled “Destin2: Integrative and Cross-Modality Analysis of Single-Cell Chromatin Accessibility Data” introduces a novel method designed for cross-modality dimension reduction, clustering, and trajectory reconstruction of single-cell ATAC-seq data. By integrating cellular-level epigenomic profiles from peak accessibility, motif deviation score, and pseudo-gene activity, Destin2 infers a shared manifold using multimodal input, followed by clustering or trajectory inference. The authors demonstrate the effectiveness of Destin2 through its application to experimental scATAC-seq datasets with both discretized cell types and transient cell states and by benchmarking against existing methods based on unimodal analyses. The findings illustrate that Destin2 corroborates and enhances existing techniques, establishing it as a valuable computational pipeline for scATAC-seq data analysis.

Another noteworthy contribution in scATAC-seq analysis is presented in the paper “Benchmarking Automated Cell Type Annotation Tools for Single-Cell ATAC-seq Data.” This study evaluates the performance of five annotation methods for identifying cell types in scATAC-seq data. By assessing classification accuracy and scalability, the authors provide valuable guidance for selecting appropriate tools for cell type annotation. Using publicly available single-cell datasets from mouse and human tissues, including brain, lung, kidney, PBMC, and BMMC, the authors found Bridge integration as the most effective and robust method, impervious to alterations in data size, mislabeling rate, and sequencing depth. While Conos is highlighted for its time and memory efficiency, its prediction accuracy was compromised. The study discusses the strengths and limitations of each method, providing a well-considered recommendation for their selection.

The emergence of single-cell multiomics technology, which captures multiple omics modalities in individual cells, has opened new avenues for comprehensive cell characterization. The paper “iPoLNG––An Unsupervised Model for the Integrative Analysis of Single-Cell Multiomics Data” presents iPoLNG, an unsupervised generative model for the integration of single-cell multiomics data, including transcriptome and epigenomic profiles. iPoLNG employs computationally efficient stochastic variational inference to reconstruct low-dimensional representations of cells and features, modeling discrete counts in single-cell multiomics data with latent factors. This model can accommodate partial information where certain cell modalities are missing and leverages GPU and probabilistic programming to ensure scalability, requiring less than 15 min to handle datasets with 20,000 cells.

Clustering and trajectory analysis of general single-cell sequencing data remain fundamental tasks, and two papers in this Research Topic contribute to this field. The paper “RobustTree: An Adaptive, Robust PCA Algorithm for Embedded Tree Structure Recovery from Single-Cell Sequencing Data” introduces RobustTree, a novel adaptive and robust PCA algorithm that extracts embedded tree structures from single-cell sequencing data. The authors demonstrate that RobustTree accurately and robustly reconstructs both continuous and discrete-state topological structures from high-noise single-cell sequencing data with complex structures.

Another paper, “Subject Clustering by IF-PCA and Several Recent Methods,” discusses subject clustering, where measured features are employed to cluster subjects into multiple groups. The authors propose IF-VAE, a novel method that combines Variational Auto-Encoder (VAE) with Influential Feature PCA (IF-PCA), and they compare IF-VAE with several other methods on gene microarray data sets and single-cell RNA-seq data sets. The findings indicate that IF-VAE significantly enhances VAE but falls short compared to IF-PCA. Interestingly, IF-PCA demonstrates competitiveness, slightly surpassing Seurat and SC3 over single-cell data sets.

The papers presented in this Research Topic represent substantial advancements in statistical and computational methods for single-cell sequencing analysis. From integrating multiple data modalities to exploring innovative frameworks for scATAC-seq data, these contributions enhance our ability to interpret and apply single-cell sequencing data effectively, ultimately deepening our understanding of cellular heterogeneity and dynamics.

However, it is important to acknowledge that the field of single-cell analysis is continually evolving, with ongoing challenges and emerging trends. Scalable and efficient tools are needed to manage the ever-increasing volumes of multi-modality single-cell or subcellular omics data.

In addition, as technology advances, various spatial transcriptomics protocols have been devised that reveal cellular omics attributes in conjunction with spatial cell locations; certain protocols can even discern subcellular structures. Consequently, there is a pressing need for novel methods to analyze data derived from these cutting-edge technologies. Additionally, promoting data sharing and collaborations among the scientific community is integral to establishing a diverse and comprehensive information repository. Continued research and refinement of analytical tools, especially tools designed to integrate multiple data types and data sources, will undoubtedly propel single-cell research forward, enabling a more nuanced understanding of biological systems.

In conclusion, the papers presented in this Research Topic demonstrate the remarkable progress made in statistical and computational methods for single-cell sequencing analysis. These advancements hold great promise for unlocking the full potential of single-cell data and accelerating discoveries in various fields, ranging from developmental biology to disease research. As the field continues to evolve, it is crucial to embrace collaborative efforts and innovative approaches to further enhance our understanding of the complexity of cellular processes and advance scientific knowledge.

